# Endothelial progenitor cells: what use for the cardiologist?

**DOI:** 10.1186/2040-2384-2-6

**Published:** 2010-02-22

**Authors:** Aurangzeb Siddique, Eduard Shantsila, Gregory YH Lip, Chetan Varma

**Affiliations:** 1 Haemostasis, Thrombosis and Vascular Biology Unit, University of Birmingham Centre for Cardiovascular Sciences, City Hospital, Birmingham, UK; 2 Department of Cardiology, City Hospital, Birmingham, UK

## Abstract

Endothelial Progenitor Cells (EPC) were first described in 1997 and have since been the subject of numerous investigative studies exploring the potential of these cells in the process of cardiovascular damage and repair. Whilst their exact definition and mechanism of action remains unclear, they are directly influenced by different cardiovascular risk factors and have a definite role to play in defining cardiovascular risk. Furthermore, EPCs may have important therapeutic implications and further understanding of their pathophysiology has enabled us to explore new possibilities in the management of cardiovascular disease. This review article aims to provide an overview of the vast literature on EPCs in relation to clinical cardiology.

## Introduction

Maintenance of endothelial integrity and functioning is vital to the preservation of a healthy vasculature [1]. Thus, the impairment as well as insufficient recovery potential of the endothelial cell monolayer is believed to be a critical factor during the initiaton and progression of atherosclerosis [2]. Indeed, endothelial damage/dysfunction has been proved to be involved in the pathogenesis of atherothrombotic vascular disease, with important prognostic and therapeutic implications.

Although the possibility that adult endothelial precursors may exist was suggested more than four decades ago, Asahara et al. first reported isolation of putative adult endothelial precursors which we now recognize as endothelial progenitor cells (EPCs), only in 1997 [3,4]. Endothelial progenitors were able to differentiate into mature endothelial cells and promote repair of damaged endothelium [5-8]. Progressively increasing number of studies has been devoted to these enigmatic cells in the recent years and their close association with multiple markers of cardiovascular health is now well-recognised.

Apart from a diagnostic and prognostic role, EPCs may be attractive target for treatment and, at the same time, be used themselves in an attempt to stimulate angiogenesis, vasculogenesis and cardiac performance. As an example, CD34 antibody coated stents designed to attract EPCs to the healing endothelium are under intensive clinical trial investigation. However, the precise role of EPCs in vascular pathology still needs to be further clarified as results of some currently available studies in the literature are controversial.

This article aims to provide an overview of the vast literature on EPCs in relation to clinical cardiology.

### EPCs ageing and physical activity

Physiological factors and conventional risk factors for atherosclerosis are associated with variations of the number and activity of endothelial progenitors and may be the bridge linking EPCs to common cardiovascular disorders such as coronary artery disease (CAD), myocardial infarction and heart failure.

EPC characteristics are closely associated with the presence of various cardiovascular risk factors (Table 1). EPC numbers or function - or both - may be affected. For example, smoking contributes towards reducing the number of circulating EPCs, whilst hypertension reduces migratory capacity [9]. Serum LDL cholesterol levels, a positive family history of CAD, and age have all been shown to influence the number and migration of circulating CD34^+ ^cells and EPCs [9].

**Table 1:** Effects of cardiovascular risk factors on EPC number and function

Cardiovascular risk factors	Effect on EPC number and function	EPC category	References
Hypercholesterolemia	Reduced EPC number, impaired EPC migratory capacity	Circulating EPCs (CD133, 34, 45) CFU-ECs	[9,23]
Diabetes Mellitus	Reduced EPC number, impaired EPC migratory capacity	Circulating EPCs (CD31, 34, KDR) CFU-ECs	[26,27,29,30]
Hypertension	Inverse relationship of EPC number with systolic blood pressure	Circulating EPCs (CD133, 34, KDR)	[9,34]
Smoking	Affects EPC number in a dose-dependent manner	Circulating EPCs (CD133, 34, KDR) CFU-ECs	[35,36]
Ageing	Reduced migration and proliferation	Circulating EPCs (CD133, 34, 45) CFU-ECs	[10-14,113]
Exercise	Increased EPC number and function	Circulating EPCs (CD133, 34, 45) CFU-ECs	[15,17-20]

EPC - endothelial progenitor cells

There is an age-related quantitative decline in bone marrow cells expressing endothelial progenitor markers [3]. Jie et al analysed the number of circulating CD34^+^/KDR^+ ^EPCs in healthy subjects aged from 1 to 81 years old, and an inverse relationship with age was observed [10]. The progressive reduction in different progenitor cell populations with age was also confirmed by Shaffer et al, both in healthy donors and in patients with peripheral arterial disease [11]. Impairment of the functional activity of endothelial progenitors also parallels downregulation of their numbers [12]. This decline in EPC clonogenic capacity appears to occur at an earlier age, followed by a decline in migratory activity.

Admittedly, some controversy still exists on the relationship of age to EPC levels. For example, Pelliccia et al failed to find any difference in absolute numbers of CD34+, CD133^+^, CD105^+^, and CD14^+ ^cells in older patients with CAD indicating a strong impact of factors other than age for the presence in atherosclerosis and re-emphasising the necessity of having a precise definition of EPC populations studied [13]. The decrease in EPC recruitment in the elderly may be associated with downregulation of tissue hypoxia-inducible factor 1 or insufficient local expression of VEGF, one of the key attractors of EPCs [14]. Furthermore, over an individual's lifetime, long-lived cells such as bone marrow endothelial precursors incur repeated exposures to oxidative stress; initially, EPCs may compensate by increasing their antioxidant responses to prevent oxidant injury. Over time, oxidant damage is likely to accumulate, thus diminishing the functional properties of EPCs.

Physical activity effectively promotes EPC health in terms of quantity, functional capacity and the prevention of apoptosis [15]. This effect is rapid and sustained, at least for 4 weeks [15]. Importantly, physical activity positively affects both bone marrow and peripheral EPC levels [15]. Furthermore, studies in animals have shown that physical activity enhances replacement of the dysfunctional endothelium by bone marrow-derived cells [16].

A rapid increase of EPC numbers following physical exertion may be attributable to acute mobilisation of the bone marrow EPC pool or a shear stress-induced release into circulation of vessel-wall resident EPCs [17,18]. Again, NO-dependent mechanisms may by involved, given that physical exercise increases NO bioavailability [19]. The effect of physical activity on EPCs may be reduced by inhibition of endogenous NO synthase [20].

### EPCs and cardiovascular risk factors

Hypercholesterolemia negatively affects both EPC number and function. Indeed, EPC count has an inverse relationship with total cholesterol and LDL-cholesterol levels [9]. Enhanced oxidative stress associated with dyslipidaemia may at least be partly involved in the dysregulation of EPC mobilisation, maturation and survival. Of note, circulating EPCs are very sensitive to oxidized LDL, resulting in premature apoptosis [21,22].

Enhanced endothelial dysfunction and damage may result in higher tissue demand for EPCs and their increased turnover. Hypercholesterolaemia may also directly affect the bone marrow, resulting in depletion or exhaustion of the bone marrow pool of endothelial progenitors, with a consequent limited supply of EPCs released into circulation [23]. Also, LDL cholesterol levels have an inverse relation with EPC migratory capacity [23]. Elevated LDL cholesterol and oxidized LDL levels impair EPC migration, via a VEGF-mediated pathway, and oxidized LDL blocks VEGF-induced EPC migration through the inhibition of NO production [24,25].

Reduced levels of EPCs have been described in both type 1 and type 2 diabetes mellitus [26]. EPC recruitment for re-endothelialization after vascular injury is impaired in patients with diabetes, and as a consequence of such EPC dysfunction, the vascular regenerative potential of this disease group may be reduced - thus, contributing to the development of vascular complications [27]. Indeed, decreased numbers and functional activity of early EPCs are significantly associated with the pathogenesis of vascular complications in either type 1 or type 2 diabetes [26,27].

Hyperglycemia significantly reduces eNOS production by EPCs with a corresponding decline in nitric oxide (NO) bioavailability [31]. The effects of high glucose could be ameliorated by co-incubation of EPCs with the NO donor sodium nitroprusside or p38 mitogen-activated protein kinase inhibitor, and deteriorated by eNOS inhibitor [28]. In contrast, antioxidants including vitamin C, N-acetylcysteine and polyethylene glycol-conjugated superoxide dismutase, and polyethylene glycol-catalase have no significant effects on EPCs [28]. This suggests that the inhibitory effects of high glucose on EPC could be reversed by NO donors, but not by various antioxidants.

Systolic blood pressure has a negative correlation with the number of circulating EPCs, but the clonogenic potential (number of CFU-ECs) is not impaired by arterial hypertension [9]. Angiotensin II accelerates the onset of EPC senescence, leading to impaired proliferation of EPCs; this seems to be inhibited by treatment with the angiotensin II type 1 receptor blocker, valsartan [29]. Ramipril also improves the proliferation and migration of EPCs, as well as *in vitro *vasculogenesis in patients with CAD [30].

These observations were confirmed in the Endothelial Progenitor Cells in Coronary Artery Disease (EPCAD) study, demonstrating that angiotensin-converting enzyme inhibitor treatment was associated with increased numbers and improved clonogenic potential of circulating EPCs, when compared with patients who were not taking angiotensin-converting enzyme inhibitors [31].

Whilst smoking leads to a reduction in EPC counts, nicotine itself may have a positive effect on EPC numbers and functional activity at low concentrations [32,33]. This is supported by the finding that the use of nicotine patches slightly increases the magnitude of the rise in EPC levels after smoking cessation [32]. However, higher nicotine levels will have cytotoxic effects on EPCs, indicating complex effects of nicotine on EPCs [33]. Indeed, the number of EPCs is reduced in chronic smokers, whilst cessation of smoking leads to rapid restoration of EPC levels [32]. In contrast, smoking cessation leads to rapid recovery of the circulating EPC population, especially amongst light smokers [32].

Recent studies have established a clear link between levels of circulating EPCs and the cumulative cardiovascular risk profile [8]. For example, Hill et al[8] hypothesized that EPCs derived from the bone marrow have a role in ongoing endothelial repair and thus, the depletion of these cells contributes to cardiovascular disease progression. Also, the number of CFU-EC colonies from peripheral blood correlates closely with endothelial function. Thus, EPC levels could be used as a 'biological marker' (or biomarker) for vascular function and the relationship to cumulative cardiovascular risk.

Although mechanisms linking cardiovascular risk factors and the impairment of EPC mobilization and function are not sufficiently well understood, the number of studies strongly indicates that chronic inflammation and oxidative stress may play a critical role, despite substantial resistance of endothelial progenitors to oxidative burden [34-36].

### EPCs and cardiovascular pathology

#### Atherosclerotic disease progression

Reduced levels of circulating EPCs have been shown to be independent predictors of atherosclerotic disease progression [37]. Indeed, endothelial integrity is a fine balance between endothelial damage and repair [37]. Since atherosclerotic risk factors are associated with reduced numbers and function of circulating EPCs, it is possible that progression of atherosclerosis is 'driven' by an impairment of EPC repairing capacity [9].

In addition, disease processes that 'damage' the endothelium per se lead to endothelial cell detachment, resulting in increased levels of circulating endothelial cells (CECs) in the blood [38]. CECs are thought to be mature cells that have detached from the intimal monolayer in response to endothelial injury and are a different cell population to EPCs [38]. There is increasing evidence to support a relation between endothelial damage/dysfunction and CECs [39,40]. An inverse relationship has been found between EPCs and CECs, as elevated numbers of CECs have been seen in patients with CAD whereas a reduction in the number of circulating EPCs has been associated with CAD [39-41].

#### Stable coronary artery disease

An inverse relationship has been found between circulating EPCs and CAD severity, independently of traditional risk factors [42] (Table 2). Indeed, patients with multivessel CAD, had significantly lower EPC counts as compared to those without - for every increase in 10 clusters of CFU-ECs, a patient's likelihood for multivessel CAD declined by 20% [42]. In contrast, Guven et al [43] showed that the number of EPCs was increased among patients with significant CAD especially in those requiring coronary intervention, and EPC numbers correlated with the maximum angiographic stenosis severity. The discrepancy between these two studies might be attributable to the degree of ischemia experienced by patients included as more severe ischemia in patients required intervention could be responcible for EPC mobilization.

**Table 2:** Effect of cardiovascular diseases on EPC number and function

Cardiovascular disorders	Effect on EPC number and function	EPC category	References
Acute coronary syndrome	Increased EPC number and function	Circulating EPC (CD34, 45,133) CFU-ECs	[45-48]
Atherosclerotic disease progression	Reduced EPC number	Circulating EPC (CD14) CFU-ECs	[37]
Severity of coronary artery disease	Reduced EPC number/Increased EPC number	Circulating EPC (CD34, 133, KDR) CFU-ECs	[34,42,43]
Heart failure	Increased EPC number at early mild heart failure, decreased EPC number in severe heart failure	CFU-ECs	[52,53]

EPC - endothelial progenitor cells

Measurement of EPCs is of predictive value for cardiovascular outcomes in stable CAD patients. For example, Werner et al measured EPCs positive for the CD34 and KDR in 519 patients with CAD confirmed on coronary angiography, and evaluated the association between baseline levels of EPCs and major adverse cardiac event rates at 12 months [34]. After adjustment for age, sex, vascular risk factors and other relevant variables, increased levels of EPCs were associated with a reduced risk of death from cardiovascular causes, a first major cardiovascular event, revascularization and hospitalization. This association was independent of severity of CAD, cardiovascular risk factors and drug therapies known to influence cardiovascular outcomes.

Of interest, EPC number and function is closely associated with coronary endothelial function. In 90 patients with CAD coronary endothelial function was assessed using an intracoronary acetylcholine with quantitative coronary angiography [44]. The number of circulating CD133^+ ^or CD34^+^/KDR^+ ^EPCs and EPC function estimated by proliferation of CFU-ECs inversely correspondent with the degree of endothelial function impairment [44]. Multivariate analysis showed that the number of EPCs predicted severe endothelial dysfunction independently of classical cardiovascular risk factors.

#### Acute coronary syndromes

Acute coronary syndromes (ACS) are associated with increased levels of inflammatory and haematopoietic cytokines, which in turn can mobilise progenitor cells from the bone marrow [45-47]. Of the many studies in this patient group, Shintani et al were the first to describe a rapid and significant increase in the CD34^+ ^EPC numbers, which reached a maximum at 7 days after the onset of ischaemia in acute myocardial infarction (AMI) [48]. Similarly, Massa et al described spontaneous mobilisation and a 5.8-fold increase of CD34^+ ^progenitor cells, which peaked about 3 hours after the onset of symptoms, significantly decreased after 7 days, and reached levels comparable with those of healthy subjects within 2 months [46]. Other cell subpopulations, such as CD34^+^/CD117^+^, CD34^+^/CXCR4^+^, CD34^+^/CD38^+ ^and CD34^+^/CD45^+ ^broadly follow the same pattern as that of EPCs [45,46]. Sufficient EPC numbers, as well as the capacity to differentiate into mature endothelial cells are considered to be essential for myocardial functional recovery and infarct size reduction after AMI [49].

Of note, the type of revascularization (ie, thrombolytic therapy or primary angioplasty) does not seem to affect EPC mobilization [50]. However, ischemia per se may be a primary factor for EPC mobilization, given a significant rise of EPC numbers in patients with unstable angina [50]. An inflammatory state, as shown by high C-reactive protein levels, is also involved in the modulation of adhesive properties of EPCs in ACS [51].

#### Congestive heart failure

Biphasic changes of the number of CD34+ cells and EPCs have been observed in patients with heart failure. EPCs are significantly up-regulated in mild heart failure (NYHA class I-II) but their mobilization is severely depressed in patients with advanced heart failure (NYHA III-IV) irrespectively of the origin of the disease [52,53]. Depletion in EPCs has been correlated with high levels of TNF-α indicating that endothelial precursors arem additional 'victim' of excessive inflammation seen in heart failure [52]. These data are in accordance with observations in animal model that statin-induced prevention of left ventricular dysfunction was strongly associated the ability of statins to mobilize EPCs [54].

The proportion of CD34+ cells in hospitalised patients with congestive heart failure increases with the improvement of clinical status and is correlated with BNP levels. Of note, Michowitz et al have recently demonstrated that the CFU-EC numbers, together with age and presence of diabetes were independent predictor of all-cause mortality in 107 patients with congestive heart failure [55].

#### Coronary artery in-stent restenosis and cardiac surgery

Restenosis after coronary artery stenting remains a significant problem in the practice of interventional cardiology [56]. Prevention of restenosis may be promoted by endothelial regeneration through the administration of growth factors, endothelial cell seeding, vessel reconstruction with autologous endothelial cell/fibrin matrix, and the use of stents designed to attract and capture EPCs [56].

Traditionally, the regeneration of injured endothelium has been believed to be due to the migration and proliferation of neighboring endothelial cells. This hypothesis has been challenged by animal studies, which reveal that transfusion of EPCs led to reduced intimal thickening of the injured arterial wall, as a result of accelerated reendothelialization [57]. Stents coated with an integrin-binding cyclic Arg-Gly-Asp peptide appear to limit coronary neointima formation and accelerates endothelialization by attracting EPCs, at least in an animal model [58].

What are the implications for therapy? The MAGIC cell trial demonstrated that G-CSF therapy with intracoronary infusion of peripheral blood stem cells improved cardiac function, and promoted angiogenesis in patients with acute myocardial infarction(AMI) [59]. At the same time, the concern over aggravation of restenosis was raised. However, the MAGIC Cell-3-DES trial showed that G-CSF - based stem cell therapy was both feasible and safe with DES implantation, eliminating the risk of restenosis [60].

Nonetheless, G-CSF administration itself has been shown to be associated with enhanced neointimal hyperplasia, possibly by the stimulation of excessive proliferation and the migration of smooth muscle cells, thus promoting re-stenosis of stented arteries [61]. Moreover, the possibility remains that EPCs may be independently responsible for stent restenosis, as a strong correlation has been shown between circulating CD34^+ ^cells and late luminal loss following coronary angiography [62]. In a multivariate regression model, a change in CD34+ cells independently predicts late lumen loss [63]. However, CD34 cells may be the ones that aggravated in stent restenosis per se. CD34 cells are not authentic EPCs but myeloid stem cells/progenitors that may differentiate into smooth muscle cells depending on the situation or in the presence of platelet derived growth factor [63].

The role of EPCs in process of post-arterial injury recovery/impairment is pretty complex. The ability of EPCs to home to arterial neointima was initially shown in a experimental minipig model [64]. Inoue et al subssequently confirmed that an increase in EPC numbers following bare-metal stent implantation in patients with CAD and the extent of EPC elevation correlated with the risk of stent restenosis [65]. In contrast, DESs (eg, sirolimus-eluting stents) are capable of preventing procedure-related EPC mobilization [65].

Rapamycin has been also shown to inhibit proliferation, migration, and differentiation of human EPCs in vitro, suggesting that the targeting of EPCs may be an additional mechanism of their successful prevention of re-stenosis by the employment of DESs [66]. Indeed, a reduction of EPC numbers may be one mechanism through which drug-eluting stents actually inhibit restenosis. Of note, cell-cycle inhibitors (sirolimus and paclitaxel) reduce neointimal formation by impeding smooth muscle cells proliferation and migration, as well as cause impairment of the normal process of healing of the injured arterial wall, leading to delayed re-endothelialization [67].

Are EPCs really a negative factor in interventional procedures? In contrast to the data where increased numbers of EPCs lead to excessive neointima hyperplasia, Matsuo et al have demonstrated that circulating EPCs in patients with in-stent restenosis grow less colonies of CFU-ECs and these cells have signs of increased senescence compared to patients with successful arterial healing [68]. Increased number of senescent EPCs were an independent predictor of stent restenosis, but these data also suggest that functional characteristics of EPCs - rather than numbers per se - may be critical for efficient re-endothelisation. However, one has to keep in mind that circulating CD34+ cells are not exclusively made up of endothelial precursors but also of hematopoietic stem cells and attraction of CD34+ cells may also involve homing of certain populations of inflammatory leukocytes to sites of vascular damage.

The numbers of circulating EPCs may be significantly affected by cardiac surgery, including coronary artery bypass grafting and valve surgery. For example, Smadja et al demonstrated selective mobilisation of endothelial progenitors after different cardiac operations [69], probably in response to tissue damage and application of cardiovascular bypass. Indeed, the mobilization of EPCs from the bone marrow may be promptly stimulated by cardiopulmonary bypass but the extent of such mobilisation is significantly affected by pre-existing risk factors and the European System for Cardiac Operative Risk Evaluation (EuroSCORE) score. Further research is needed to establish the dynamics of EPC during or following cardiac surgery, and how this affects prognosis [70].

### Therapeutic modification of EPCs

#### Statins

Statin therapy is associated with an increase in the number of circulating EPCs in patients with CAD [20] (Table 3). The increase in EPCs and improvement of their migratory capacity was significant as early as at 1 week after the initiation of treatment with atorvastatin, with a 3-fold increase at 3 to 4 weeks of therapy [20]. The mobilization of circulating EPCs, along with their enhanced functional activity may contribute to the beneficial effects of statins in patients with CAD [20]. The migration and incorporation of EPCs to the sites of re-endothelialization was found significantly increased after statin administration [20,71].

**Table 3:** Effect of drug treatments on EPC number and function

Medication	Effect on EPC number and function	Reference
Atorvastatin	Increased EPC number and migration	[20]
Simvastatin, Mevastatin, Atorvastatin	Increased proliferation	[114]
Simvastatin	Increased adhesion	[69]
Atorvastatin, ezetimibe	Increased EPC number	[115]
Valsartan	Reduced senescence	[32]
Ramipril	Increased EPC number, proliferation, migration, adhesion, in vitro vasculogenesis capacity	[33]
Rosiglitazone	Increase in EPC culture and migration	[116]

While short-term treatment with statins has been seen to increase both EPC number and function, more long-term treatment with statins may have effects to the contrary. Indeed, there is a biphasic effect of statins on EPC counts. In one study of 144 patients with angiographically documented CAD, the administration of statins for more then 4 weeks resulted in a significant decrease of EPC numbers, as determined by flow cytometry and *in vitro *culture [72]. However, only CFU-ECs were evaluated in this study, and as previously discussed, this special subpopulation of EPCs is probably monocytic in origin, with limited capacity to be incorporated into the microvasculature, but may still promote angiogenesis by the release of proangiogenic factors [73]. These observations were further substantiated by Deschaseaux et al [74], who demonstrated that statin-induced depression of EPCs was only attributed to CFU-ECs, whereas the ECFC populations, were preserved or even enhanced in the circulation by long-term statin treatment.

In summary, the effects of statins on EPCs are complex. They help to mobilize early EPCs, which is especially important in acute ischaemic conditions such as AMI, but strong long-term positive effects of statins may be partly explained by augmentation of ECFC, which are believed to be 'true EPCs' responsible for vasculogenesis.

#### ACE inhibitors and Angiotensin II receptor blockers

Angiotensin II subtype 1-receptor blockade increases the number of EPCs, an effect which seems to be common to all angiotensin II receptor antagonists [75]. VEGF appears to be involved in angiotensin receptor blocker-mediated EPC stimulation [32].

Min et al showed that treatment with ramipril 5 mg daily for 4 weeks in patients with CAD was associated with an approximately 1.5-fold increase in the number of circulating EPCs within 1 week of initiating treatment [33]. This trend persisted with increased levels to approximately 2.5-fold throughout the 4-week study period. In addition, increases in the functional activity of EPCs - as assessed by their proliferation, migration, adhesion and in vitro vasculogenesis capacity - was also seen. Broadly similar effects were also seen with enalapril, as shown in a study by Wang et al, whereby patients on enalapril displayed a significant increase in circulating EPCs in response to ischemic stress [76]. Enalapril also caused a 6-fold increase in the contribution of bone marrow-derived EPCs to the ischemia-induced neovascularization.

#### Other therapeutic approaches

Administration of the PPAR-gamma agonists has been associated with elevation of EPC numbers and improvement of their function [77]. For example, rosiglitazone was shown to attenuate the negative effects of C-reactive protein on EPCs and enhance NO-production of EPCs [78]. Other available data indicate that positive effects of PPAR-gamma agonists on EPCs may be one of mechanisms on the cardiovascular system [78,79]. In addition to the effects of PPAR-gamma agonists on EPCs, another PPAR subtype -the PPAR agonists- has recently attracted attention. For example, Han et al showed PPAR agonists modulate CFU-EC promoting vasculogenesis [80]. There are also provasculogenic effects of PPAR agonists on ECFCs [81].

Oestrogens augment production and survival of EPCs, thereby increasing the circulating levels of these cells [82]. Moreover, oestrogens not only increase EPC count, but also their ability to effectively home to the sites of vascular lesions [82]. Oestradiol may enhances EPC mobilization though NO-mediated pathways [83].

#### Implantation of CD34 antibody coated stents

Treatment options for CAD include the mechanical dilatation of one or more areas of narrowing within the coronary artery using percutaneous coronary intervention (PCI). The original 'balloon-only' technique was improved by the use of bare-metal stents (BMS). However, BMSs are still prone to restenosis in up to 30% of patients [84]. To reduce stent restenosis, the use of DESs which can elute antimitotic medications has become widespread. However, DES use has been associated with late stent thrombosis [85,86]. Indeed, the risk of stent thrombosis with DES is greater than that seen with BMS due to impaired endothelial healing and delayed endothelialisation [87].

In contrast, endothelialisation with a third type of stent, the CD34 antibody coated stents based on EPC capture technology, usually occurs within 7 days in animal studies [88]. Rapid endothelialisation and restoration of endothelial function may have several advantages, including reduced restenosis and reduced stent thrombosis rate. In addition, early cessation of dual antiplatelet therapy may be possible, reducing the inherent risk of this.

Effectiveness of CD34 covered stents has been tested in a series of studies performed under the HEALING (Healthy Endothelial Accelerated Lining Inhibits Neointimal Growth) program. The first of these studies was the Healthy Endothelial Accelerated Lining Inhibits Neointimal Growth-First In Man (HEALING-FIM) Registry which demonstrated feasibility and safety of EPC capture by CD34 covered stents in stable CAD [89]. Subsequently, the HEALING II and the HEALING III studies, and ultimately the real-world e-Healing multi-center registry of the Genous™ pro-healing EPC capturing stent (OrbusNeich, Netherlands) have been performed.

The e-Healing registry established that the rates of cardiac death, MI and target lesion revascularization with the CD34 covered stents were low and comparable to the early Taxus registry studies [90]. These stents were also tested for use in primary PCI in patients with AMI. Implantation of these stents was associated with relatively low rate of major adverse cardiac events: 1.6% in-hospital, 4.2% at 30 days, 5.8% at 6 months, and 9.2% at 1 year; there was 1 patient each with acute and subacute stent thrombosis but no incidence of late stent thrombosis [91]. When used in 'high risk' patients (33% with diabetes, 73% with ACS, 8% with left ventricular dysfunction, 9% with multivessel intervention, 56% type B2/C lesions) 14 month follow-up revealed an event rate of 13% for noncardiac death and AMI, 13% for repeated percutaneous revascularization and 4% for de novo lesions [92]. No bypass surgery was preformed. The study showed that the CD34-covered stents were safe and effective, with satisfactory immediate results and mid-term outcome, without evidence of stent thrombosis

In a recent report from the ongoing multi-centre, randomized the Tri-stent adjudication (TRIAS) study CD34-covered stents were as effective as paclitaxel-eluting stents for the prevention of re-stenosis during 12-month follow-up; however, 4 cases of out-of-hospital thrombotic events were reported for paclitaxel-covered stents, whilst no such events were reported in patients with implanted CD34-covered stents [93].

### Transplantation of EPCs

The available data that demonstrate angiogenic properties of EPCs and favorable outcomes of animal studies have encouraged clinical trials in patients with ischemic heart disease, particularly in the AMI setting [94]. However, results of human studies have proved to be controversial with some trials reporting significant improvement in cardiac vascularisation and performance while others failing to show any benefits. Results of randomized clinical studies are on cardiac transplantation of cells with endothelial progenitor potential are summarized in table 4.

**Table 4:** Randomized clinical studies on transplantation of cells which include endothelial progenitors

Study/year	Disorder	N	Delivery route	Cells delivered	Follow-up (months)	Effectiveness
Strauer et al. (2002) [117]	AMI	20	Intracoronary	BM-MNCs	4	Effective
Kang et al. MAGIC (2004) [92]	AMI Old MI	27	Intracoronary	PB-MNCs and G-CSF	6	Effective, but high rate of in-stent re-stenosis
Schachinger et al. TOPCARE-AMI (2004) [118]	AMI	59	Intracoronary	PB-MNCs or BM-MNCs	12	Effective
Ruan et al. (2005) [119]	Acute MI	20	Intracoronary	BM-MNCs	6	Effective
Strauer et al. IACT (2005) [120]	Chronic CAD	36	Intracoronary	BM-MNCs	3	Effective
Bartunek et al. (2005) [111]	Recent MI	35	Intracoronary	CD133+	4	Effective
Erbs et al. (2005) [112]	Chronic CAD	26	Intracoronary	Cultured PB-EPCs	3	Effective
Assmus et al. (2006) [111]	Chronic CAD	53	Intracoronary	BM-MNCs	3	Effective
Meyer et al. BOOST (2006) [104]	Acute MI	60	Intracoronary	BM-MNCs	18	Effective at 6 month, ineffective at 18 month
Hendrikx et al. (2006) [121]	Heart failure	20	Intramyocardial	BM-MNCs	4	Ineffective
Janssens et al. (2006) [105]	MI	67	Intracoronary	BM-MNCs	4	Ineffective
Kang et al. MAGIC Cell-3-DES (2006) [60]	Acute MI Old MI	82	Intracoronary	PB-MNCs and G-CSF	6	Effective
Schachinger et al. REPAIR-AMI (2006) [106,122]	AMI	201	Intracoronary	BM-MNCs	12	Effective
Ge et al. TCT-STAMI (2006) [95]	MI	20	Intracoronary	BM-MNCs	6	Effective
Meluzin et al. (2007) [122]	Acute MI	60	Intracoronary	BM-MNCs	12	Effective

AMI - acute myocardial infarction, BM - bone marrow, CAD - coronary artery disease, G-CSF - granulocyte-colony stimulating factor, MI - myocardial infarction, MNC - mononuclear cell, PB - peripheral blood.

The majority of completed randomized trials have demonstrated some benefits of stem cells treatment, and studies uniformly report the safety of this approach with no specific adverse events observed (including proarrhythmia, oncology or excessive inflammatory burden)[95,96]. High rate of in-stent restenosis in the MAGIC study where cell therapy was combined with G-CSF administration has been discussed above [59]. In the BOOST trial, intracoronary implantation of bone marrow cells did not provide long-term benefit on left ventricular systolic function after AMI compared to a randomized control group; however, stem cell therapy was associated with acceleration of left ventricular recovery [97].

In the study by Janssens et al [98], transfer of bone marrow stem cells to the coronary artery in 67 patients with AMI did not contributed to improvement of the global left ventricular function, but did favorably affected infarct remodelling at 4 months follow-up, with a reduction in infarct size. In the double-blind, placebo-controlled multicentre REPAIR-AMI trial, 204 patients with MI were randomized to bone marrow-derived cells or placebo [99]. At 12 months, the pre-specified cumulative endpoint of death, myocardial infarction, or necessity for revascularization was significantly reduced in the stem cell-therapy group compared with placebo; of note, stem cells therapy was an independent predictor of a favorable clinical outcome in this study [99].

Alternative to intracoronary infusion routs of stem cell delivery such as intramyocardial implantation (either during cardiac surgery on NOGA system) have also been tested [100,101]. In the only randomised with percutaneous transendocardial injections of CD34+ cells to patient with severe intractable angina, this method was found to be feasible and safe and is being extended into a larger ongoing phase IIb study [102].

Given the controversy of trial results, important issues have arisen about the factors affecting the efficacy of such therapy. In addition to route of administration, the time of stem cell delivery (ie. after AMI), origin and number of cells used may be critical. In fact, the majority of studies have used unselected mononuclear cells with unknown but evidently very low proportion of endothelial progenitors but with progenitors of other origins (eg. mesenchimal stem cells), this could be potentially favorable for cardiac recovery. Indeed, when either bone marrow or circulating mononuclear cells were delivered to infarct-related coronary arteries, treatment with bone morrow cells resulted in a better improvement of left ventricular contractility, when compared to peripheral blood cells [103]. Several studies with selected CD34+, CD133+ cells or cultured peripheral blood EPCs have been successfully performed but large controlled studies are required to evaluate their clinical utility [102,104,105].

Although different factors may impair EPCs-mediated vascular repair (for example, abnormality in their mobilisation from bone marrow and homing to the damaged vascular tissues, and exhaustion of their bone marrow niche), the available data strongly indicate functional characteristics of circulating endothelial progenitors are relevant. Accordingly, appropriate genetic modification of EPCs before their implantation may be a way to improve their angiogenic potential. The feasibility of this approach has been demonstrated by genetic inhibition of glycogen synthase kinase-3β signaling in human EPCs that was associated with significant enhancement of their angiogenic properties in an animal model of ischaemia [106]. Additionally, the angiogenic potential of EPCs can be improved by non-genetic ex-vivo stimulus (for example, by exposure to hypoxia). However, the clinical relevance of these approaches needs further investigation [107].

### Understanding of EPCs - where we are now?

Despite more that a decade of very intensive research and many studies devoted to the problem of understanding EPC biology, their potential clinical role is still largely limited by lack of a consensus on the phenotypic and functional definition of endothelial precursors [108]. At present, a single term 'EPC' refers to a very diverse group of cells of different lineages which appear to have some angiogenic potential, but not necessarily the ability to differentiate into functional endothelial cells, as expected from their name. Accordingly, a booming number of new publications on 'EPCs' may have limited scientific impact without a clear understanding what type of cell is actually being analysed.

Currently only so-called 'outgrowth endothelial cells' (or 'late' EPCs) are known to uniformly give origin to functional endotheliocytes. Indeed, CD34 or CD133 and alone or in different combinations (often with KDR) are the most popular markers used to define 'circulating EPCs'. This popularity stems from initial reports showing that CD34+/KDR+ cells could form endothelium-like cells in vitro [4,109]. Although many studies have employed CD34+/CD133+/KDR+ cells as their definition of 'true EPCs' their ability to generate endothelial cells has never been reliably proved. Furthermore, these cell populations probably represents a subset of CD45+ haematopoietic progenitors but do not form endothelial cells in vitro [110]. Inclusion of any additional markers in the definition of 'EPC' may hamper a holistic approach towards EPC analysis even further and should only be based on robust data confirming the functional identity of the cells analysed.

The problem with the identification of functional EPCs is also present with approached based on cell culture. For example, markers previously used to prove endothelial identity of putative progenitors, such as CD31, lectin binding or LDL accumulation are now known to be non-specific for the endothelial lineage, but also characteristic for cells of haematopoietic origin] [111,112]. In fact, the majority of so called 'early' EPCs represent populations of monocytes and lymphocytes which co-express 'endothelial' markers and possess some degree of angiogenic capacity [36]. This reemphasises that the initiation of any new clinically-relevant study on EPCs should only be based on clear understanding of type of cells being analysed.

## Conclusion

Since the discovery of EPCs, there has been a rapid proliferation of research data on the relation of EPC to cardiovascular risk, pathology and treatment. So far, EPCs have been implicated in the whole cardiovascular disease process, and many conventional therapies have been shown to alter EPC number and function. More recently, attempts to utilise the clinical potential of EPCs such as in the form of CD34-antibody coated stents, has been attempted. Further challenges will be to develop simple techniques to measure EPCs numbers and function accurately and quickly, as these cells may help determine cardiac risk and outcomes for patients with heart disease.

## Competing interests

AS is funded by a research grant from Orbus Neisch (Orbus Neisch, Netherlands). ES is funded by a research grant of the Heart Failure Association of European Society of Cardiology. CV is UK national coordinating investigator for the TRIAS programme. GL and CV are both investigators in the TRIAS trials.

## Authors' contributions

AS - selected publications for the review, drafted manuscript; ES - participated in the design of the review, drafted manuscript; GYHL - designed manuscripts, edited manuscript; CV - edited manuscript. All authors read and approved the final manuscript.

## Abbreviations

ACS: acute coronary syndrome; AMI: acute myocardial infarction; BMS: bare-metal stents; CAD: coronary artery disease; CEC: circulating endothelial cells; CFU-EC: colony forming unit-endothelial cell; ECFC: endothelial colony forming cells; eNOS: endothelial nitric oxide synthase; EPC: endothelial progenitor cell; G-CSF: granulocyte-colony stimulating factor; HUVEC: human umbilical vein endothelial cells; KDR: kinase insert domain receptor; NO: nitric oxide; PCI: percutaneous coronary intervention; TERT: telomerase reverse transcriptase; TRF: telomere repeat-binding factors; VEGF: vascular endothelial growth factor
